# Differences in responses of invasive and native plants to climate change: a case study of *Bidens* (*Asteracea*) from China

**DOI:** 10.3389/fpls.2025.1583552

**Published:** 2025-06-30

**Authors:** Haiyan Xiao, Da Liao, Shujian Zhang, Yuxin Zhang, Omer Elnour Rehab, Jianjun Zeng, Xiaohong Yan, Qitao Su, Bing Zhou

**Affiliations:** ^1^ Key Laboratory of Jiangxi Province for Biological Invasion and Biosecurity, School of Life Sciences, Jinggangshan University, Ji’an, China; ^2^ Applied College, King Khalid University, Dhahran Aljanob, Saudi Arabia

**Keywords:** climate change, *Bidens*, MAXENT model, distribution pattern, adaptability

## Abstract

Global warming has emerged as one of the most pressing environmental challenges. Concurrently, plant invasion has been exacerbated by ongoing climate change, posing a severe ecological threat. This study investigates the distribution patterns of both invasive and native species within the *Bidens* genus and their responses to projected climate change. The MaxEnt model, was used to predict the potential distribution ranges under both current and future climate conditions. The results showed a distinct difference in suitable area distributions between invasive and native species. Under future climate scenarios, most studied species (except *B. pilosa*, *B. maximowicziana*, and *B. radiata*) showed an expansion in their suitable habitats. Notably, we observed a latitudinal migration pattern in *Bidens* species distribution, with invasive species primarily influenced by precipitation during the warmest quarter, while native species were more affected by anthropogenic factors. These results underscore the need for enhanced public awareness of invasion risks and the establishment of dedicated protection zones for both invasive and native species. This study provides critical insights into the potential distribution patterns of *Bidens* species under climate change. It also, offers valuable scientific support for development of invasive species management strategies and native species conservation mechanisms.

## Introduction

Global warming has become a serious challenge for core productivity,food security, and ecosystem sustainability. The phenomenon manifested by increase in global temperature and resulting in increase in the frequency of extreme climate events, such as droughts, and floods (Byeon et al., 2018). In recent decades, man-made greenhouse gas emissions have led to an increase in global average temperature (Nikendel et al., 2020; Cook et al., 2013; Hayat et al., 2024). With the advancement of globalization, plant invasion has become a serious challenge, causing significant ecological, social and economic losses (Zhang et al., 2022a). Climate change is one of the driving factors of alien invasive plants (Zhang et al., 2024). The driving forces for plant expansion or regeneration are precipitation, light, temperature, etc. To cope with climate change, species will change their distribution to adapt to the new environment, resulting in the migration of species to suitable areas (Zhang et al., 2020). In general, global warming forces species to migrate to high latitudes and high altitudes to survive (Wilson et al., 2007). Xu et al. (2023) found that global warming has changed the trend of the northward migration of the invasive grass *Cenchrus alopecuroides* (L.) Thunb. in the suitable area of China. Cao et al. (2018) showed that the response of invasive plants to climate change has greater phenological plasticity, and it aggravate the invasion of invasive plants and accelerate their invasion process. At the same time, human activities are also an important factor affecting the distribution of species, which directly affects the spatial distribution and diversity of plants (Zhao et al., 2023a). For instance, Xu et al. (2024) found that climate change promoted the growth of *Aconitum leucostomum* Vorosch., while human activities inhibited the spread of *A. leucostomum*. Invasive plants can spread to new areas through human activities (Rew et al., 2018). Therefore, using human activities as predictors can more accurately show the potential distribution range and spatial pattern of future species.

The species distribution model (SDM) analyzes the actual distribution area and potential geographical location of species through the actual distribution range and environmental variables of species (Cao et al., 2018), The prediction accuracy of model training data usually increases with the increase in number of variables until the asymptote is reached. The number of variables should be less than the number of species records (Neftalí et al., 2021). At present, there are many species distribution prediction models, including MaxEnt (Phillips et al., 2006), ENFA (Hirzel et al., 2002), GLM (Nelder and Wedderburn, 1972) and GARP (Stockwell and Noble, 1992). Among them, the MaxEnt model is the most popular and most widely used species distribution model (Merow et al., 2013; Barbosa and Schneck, 2015), This model uses machine learning methods to use the existence species records to evaluate the possibility of events. Even if some species data are lacking or the sample size is small, it can maintain a high degree of accuracy and stability (Tang et al., 2021a). It has the advantages of fast calculation speed and flexible operation (Dong et al., 2023). Therefore, the MaxEnt model has become an ideal prediction tool for analyzing distribution or potential distribution (Zhang et al., 2021b). Moreover, SDM has been widely used in species conservation (Ouyang et al., 2022), invasive species prevention (Wang et al., 2023), and the distribution of endangered and threatened species (Su et al., 2024a, b; Hu et al., 2022). Su et al. (2024b) used the maxent model to analyze the migration pattern of *Hydrocera triflora* (L.) Wight & Arn. These authors found that this model provides a theoretical basis for the introduction and scientific protection of this species. Yang et al. (2023) used this model to analyze the distribution pattern of 31 Asteraceae invasive species, and provided early countermeasures for reducing the risk and impact of biological invasion. Zhang et al. (2023) used MaxEnt and random forest (RF) models to predict the potential distribution areas of *Populus euphratica* Olivier and *Tamarix chinensis* Lour. in the lower reaches of the Heihe River (40°32′N–42°39′N, 97°36′E–102°8′E).

*Bidens* L. is a genus within Asteraceae that includes approximately 230 herbaceous species around the world, which is widely grown in tropical and subtropical regions of Asia, American Continent and other continents (Deng et al., 2019). There are 12 species in China. *Bidens alba* (L.) DC., *Bidens bipinnata* L., *Bidens biternata* (Lour.) Merr. & Sherff, *Bidens cernua* L., *Bidens frondosa* L., *Bidens maximowicziana* Oett., *Bidens parviflora* Willd., *Bidens pilosa* L., *Bidens radiata* Thuill., *Bidens subalternans* DC. *Bidens tripartita* L., *Bidens vulgata* Greene, among these, *B. alba*, *B. bipinnata*, *B. frondosa*, *B. Pilosa*, *B. vulgata*, *B. subalternans* are noteworthy for being invasive species. are widely distributed and recorded in all provinces of China (Wang et al., 2020), It is mainly distributed in southern and northern China, and is born in villages, roadsides and wasteland. The heteromorphic achenes produced by the mature capitate inflorescence of *Bidens* have high yield, and have barbed spines, which are easy to adhere to the carrier and be carried and spread (Brändel, 2004).

Most of the species in the genus are often used in traditional Chinese medicines, with potential to heat, analgesia and anti-inflammation, and promote blood circulation (Fotso et al., 2014; Wang et al., 2018). Therefore, the current research on *Bidens* species only focuses on the development of taxonomic and medicinal value (Brandão et al., 1997; Chiang et al., 2004). In recent years, a variety of invasive plants have emerged in China. Most plants of *Bidens* have the characteristics of fast growth and development, large number of fruits, easy spread and diffusion. These characteristics are conducive for expanding the distribution area and occupying the residence, so as to achieve rapid invasion (Qiang and Zhang, 2022; Salgado et al., 2024). In order to scientifically clarify the distribution of *Bidens* and its response to future climate change, this study used MaxEnt model and ArcGIS V10.8 software to simulate and predict the potential distribution of *B. alba*, *B. bipinnata*, *B. frondosa*, *B. pilosa*, *B. biternata*, *B. cernua*, *B. maximowicziana*, *B. parviflora*, *B. radiata*, *B. tripartita* (four invasive species and six native species) in China in the current and future 2050s (2041-2060) and 2090s (2081-2100), and answered the following scientific questions:(1)What are the main climate factors affecting the distribution of invasive species and native species of *Bidens*? (2) How the suitable area of invasive species and native species of *Bidens* will change in the future? (3) Is the expansion trend in the distribution pattern of invasive species and native species similar under current and future climate change scenarios?

## Materials and methods

### Acquisition and processing of distribution data of *Bidens*

A total of 12 species of *Bidens* distributed in China were collected. In this analysis, *B. alba*, *B. bipinnata*, *B. biternata*, *B. cernua*, *B. frondosa*, *B. maximowicziana*, *B. parviflora*, *B. pilosa*, *B. radiata*, *B. tripartita* 10 species were used, However, two species, including *B. vulgata* and *B. subalternans* were excluded because *B. vulgata* had only two records and *B. subalternans* had only four records. Species distribution data were sourced from GBIF (https://www.gbif.org/), China Digital Herbarium (https://www.cvh.ac.cn/) and China Plant Image Library (https://ppbc.iplant.cn/), and the distribution data with specific latitude and longitude points were selected. Microsoft excel was used to process the distribution of data and saved as CSV format to remove information such as duplication and no coordinate points. ENMTools was used to remove redundant distribution data and avoid over-fitting of the model. Only one distribution record was retained in every 2km grid (Warren et al., 2010). and effective distribution data were finally obtained for analysis.

### Sources of environmental data

The environmental climate data were derived from the World Climate Database (https://www.worldclim.org/), the spatial resolution was 2.5 arc min (Fick and Hijmans, 2017), and 19 climate data (Bio1-Bio19) in the CCSM4 global climate model were downloaded from the database (**Table 1**). CMIP6 was used to release the current and future 2050s (2041-2060), 2090s (2081-2100) two time periods and SSP1-2.6, SSP2-4.5, SSP5-8.5 three climate scenarios. The SSP126 scenario was a low greenhouse gas emission condition, the SSP245 scenario was a medium greenhouse gas emission condition, and the SSP585 scenario was a high greenhouse gas emission condition. The slope and slope direction were extracted from DEM digital elevation data with an accuracy of 25 m, which were obtained from the Computer Network Information Center of the Chinese Academy of Sciences and the International website of Scientific Data (http://www.gscloud.cn/). Human active (HA) data were obtained from the Socioeconomic Data and Applications Center (SEDAC: http://sedac.ciesin.columbia.edu/wildareas/).

**Table 1:** Environmental factors information.

Variable	Description
Bio1	Annual Mean Temperature
Bio2	Mean Diurnal Range (Mean of monthly (max temp - min temp))
Bio3	Isothermality
Bio4	Temperature Seasonality
Bio5	Max Temperature of Warmest Month
Bio6	Min Temperature of Coldest Month
Bio7	Temperature Annual Range (BIO5-BIO6)
Bio8	Mean Temperature of Wettest Quarter
Bio9	Mean Temperature of Driest Quarter
Bio10	Mean Temperature of Warmest Quarter
Bio11	Mean Temperature of Coldest Quarter
Bio12	Annual Precipitation
Bio13	Precipitation of Wettest Month
Bio14	Precipitation of Driest Month
Bio15	Precipitation Seasonality (Coefficient of Variation)
Bio16	Precipitation of Wettest Quarter
Bio17	Precipitation of Driest Quarter
Bio18	Precipitation of Warmest Quarter
Bio19	Precipitation of Coldest Quarter
Aspect	Aspect
Elev	Elevation
HA	human activity factor
Slope	Slope

### Accuracy test of model construction

To perform base map analysis China map on the website of the National Bureau of Surveying and Mapping (https://nfgis.nsdi.gov.cn) was downloaded. Using MaxEnt model, the screened environmental factors were used for modeling. Then they, used to predict the suitable area of *Bidens* plants under the current climate model. Through the Jackknife method, 25% of the distribution data were randomly selected as the test data, 75% selected as the training data, while other parameters were defaulted to determine the importance of each environmental factor (Li et al., 2020a; Qiu et al., 2023). The receiver operating characteristic curve (ROC) of all environmental variables was set and calculated, and the area under the curve (AUC) was used to test the prediction results of the model. This value was considered to be one of the evaluation criteria for the prediction results of species distribution models with high reliability and wide application, and, its value is between 0-1, The value of AUC ≤ 0.60 represents failure; 0.60< AUC ≤ 0.70 indicated poor accuracy; 0.70 < AUC ≤0.80 means that the accuracy is general; 0.80< AUC ≤ 0.90 means high accuracy; 0.90 < AUC≤ 1.0, represents a very high accuracy, and the higher the AUC, the more accurate the model (Yang et al., 2023).

### Classification of suitable areas

The output of the model was imported into ArcGIS, and the conversion tool was used to convert it from asc format to raster data, and the suitable area was divided. To determine the suitable area and unsuitable area of the species, the MaxEnt output file was reclassified using the 10 percentile training existence logic threshold (TH) (Qiu et al., 2023). By combining the average value of the 10 percentile training existence logic threshold (TH) output of MaxEnt with the IPCC classification criteria, the potential habitats were divided into three categories: unsuitable area (<TH), suitable area (TH-0.66) and highly suitable area(>0.66) (Shi et al., 2021).

### Analysis of spatial pattern change and distribution centroid of species suitable area

In ArcGIS, the suitable area was binarized, and the area with distribution probability < TH was set as the unsuitable area, and the assignment was 0. The distribution probability ≥ TH area was set as the suitable area, assigned to 1, and the non-suitable/suitable binary map matrix of each period was obtained. Define 0-0 as the unsuitable area, 0-1 as the new suitable area, 1-0 as the lost suitable area, and 1-1 as the reserved suitable area. The area change, change trend and range of *Bidens* plants under different climatic scenarios and contemporary under different suitable grades were calculated, and the area and geographical range of its increase, retention and loss were obtained.

Based on the above binary map, the SDM toolbox was used to calculate and simulate the geometric center position changes of the potential suitable areas in different periods, Further, it also compares the overall change trend of the core suitable areas of *Bidens* in different periods, and reflects the influence of environmental changes on its distribution in different periods.

## Results

### Accuracy and evaluation of the model

The ROC curve analysis method was used to verify the prediction results of the potential suitable area distribution of *Bidens* genu in the current climate by the receiver operating characteristic curve (ROC). The average AUC values of the 10 repeated training sets and test sets of *Bidens* genu was greater than 0.9, indicating that the model has high stability and accurate. Therefore, it can be used to predict the potential distribution of *Bidens* genu (**Table 2**).

**Table 2:** Effective distribution records and AUC values of *Bidens* genu.

Classification	Species	Distribution Records	Test AUC	Training AUC
invasive species	*B. alba*	257	0.9866	0.9876
	*B. bipinnata*	490	0.975	0.9747
	*B. frondosa*	229	0.9875	0.9779
	*B. pilosa*	1109	0.9534	0.9551
native species	*B. biternata*	443	0.9722	0.9755
	*B. cernua*	86	0.9742	0.9822
	*B. maximowicziana*	32	0.9441	0.9953
	*B. parviflora*	415	0.9732	0.9789
	*B. radiata*	22	0.9982	0.9928
	*B. tripartita*	615	0.9643	0.9671

### Dominant environmental factors affecting distribution

**Figure 1:**
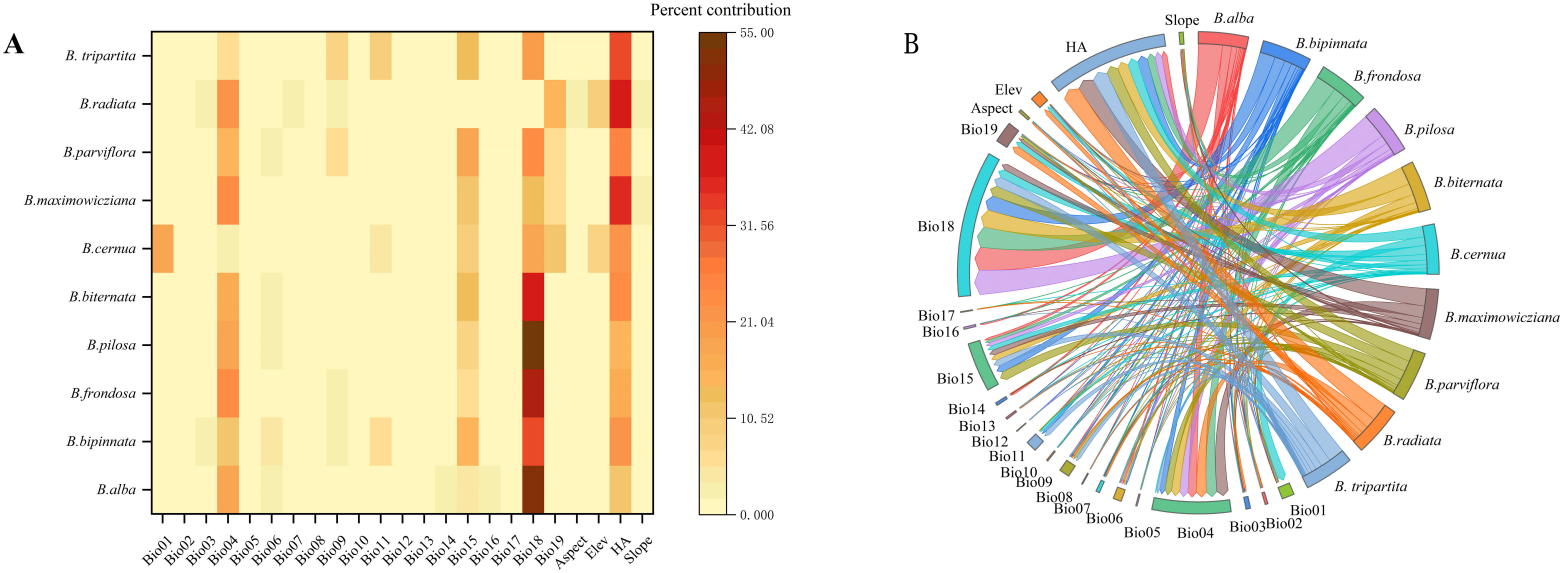
Correlation heat map **(A)** and chord diagram **(B)** of environmental factor contribution.

The MaxEnt model judges the weight of each environmental factor by the Jacknife method. According to the ranking (**Figure 1**, **Table 3**), the following table lists the three environmental factors with the highest contribution rate for each *Bidens* genu. Finally, one environmental factor with the highest contribution rate to the model generation was selected from each *Bidens* species, The dominant environmental factor of the four invasive species was Bio18 (Precipitation of Warmest Quarter). Except that the dominant environmental factor of *B. biternata* is Bio18 (Precipitation of Warmest Quarter), the dominant environmental factor of the other five native species was HA (human activity factor). The results showed that the environmental factors affecting the distribution of the most species were Bio18 (Precipitation of Warmest Quarter) and HA (human activity factor), indicating that precipitation and human activity factor were the main factors affecting the establishment of the distribution model of *Bidens* genu.

**Table 3:** The main environmental variables affecting the distribution of *Bidens* genu (top 3).

Classification	Species	Variables	Percent contribution (%)	Suitablity More Than 50%
invasive specie	*B.alba*	Bio18	50.4	511.368-1257.828mm
		Bio04	19	487.940-722.598
		HA	11.5	35.088-227.256
	*B.bipinnata*	Bio18	32.6	346.632-845.988mm
		HA	21.8	39.372-207.672
		Bio15	15.1	64.201-142.416mm
	*B.frondosa*	Bio18	44.3	403.26-598.884mm
		Bio04	23	739.359-1001.953
		HA	17	33.864-164.22
	*B.pilosa*	Bio18	52.6	459.888-1438.008mm
		Bio04	17.8	443.243-851.101
		HA	14.5	33.864-202.776
native species	*B.biternata*	Bio18	37.3	398.112-923.208mm
		HA	23.7	39.984-206.448
		Bio04	17.5	549.398-1068.998
	*B.cernua*	HA	21.9	31.416-197.88
		Bio18	18.3	300.3-815.1mm
		Bio01	18.3	2.211-12.716°C
	*B.maximowicziana*	HA	36.6	41.82-149.532
		Bio18	13.5	305.448-598.884mm
		Bio04	24.5	1186.327-1733.863
	*B.parviflora*	HA	25.8	41.208-168.504
		Bio18	22.8	300.3-567.996mm
		Bio15	18.7	89.621-136.55mm
	*B.radiata*	HA	38	33.864-170.34
		Bio04	21.1	1163.979-2057.915
		Bio19	14.5	3.354-23.481mm
	*B. tripartita*	HA	32.8	36.312-117.708
		Bio18	20.7	362.076-856.284mm
		Bio15	13.1	66.645-135.083mm

It is generally believed that when the probability of species existence is higher than 50%, the corresponding environmental factors are suitable for plant growth (Liu et al., 2020; Meng et al., 2020). For example, when the precipitation of Warmest Quarter reaches 511.368 mm, the existence probability of *B. alba* is 50%, and the precipitation of Warmest Quarter reaches 845.988 mm, the existence probability of *B. alba* is the largest, reaching 67.228%, Thereafter, it decreases with the increase of Precipitation of Warmest Quarter. When it reaches 1257.828 mm, the existence probability of *B. alba* decreases to 50%. When the Precipitation of Warmest Quarter is greater than 1257.828 mm, the probability of *B. alba* is less than 50%. Therefore, the Precipitation of Warmest Quarter between 511.368-1257.828 mm is suitable for the growth of *B. alba*. According to the response curve of *Bidens* plants to environmental factors, the range of dominant factors affecting species was calculated. The results showed that the Precipitation of Warmest Quarter in the range of 346.632 mm-1438.008 mm and the human activity factor index in the range of 31.416-197.88 were suitable for the growth of *Bidens*.

### Prediction of potential suitable areas of *Bidens* in the current situation

**Figure 2:**
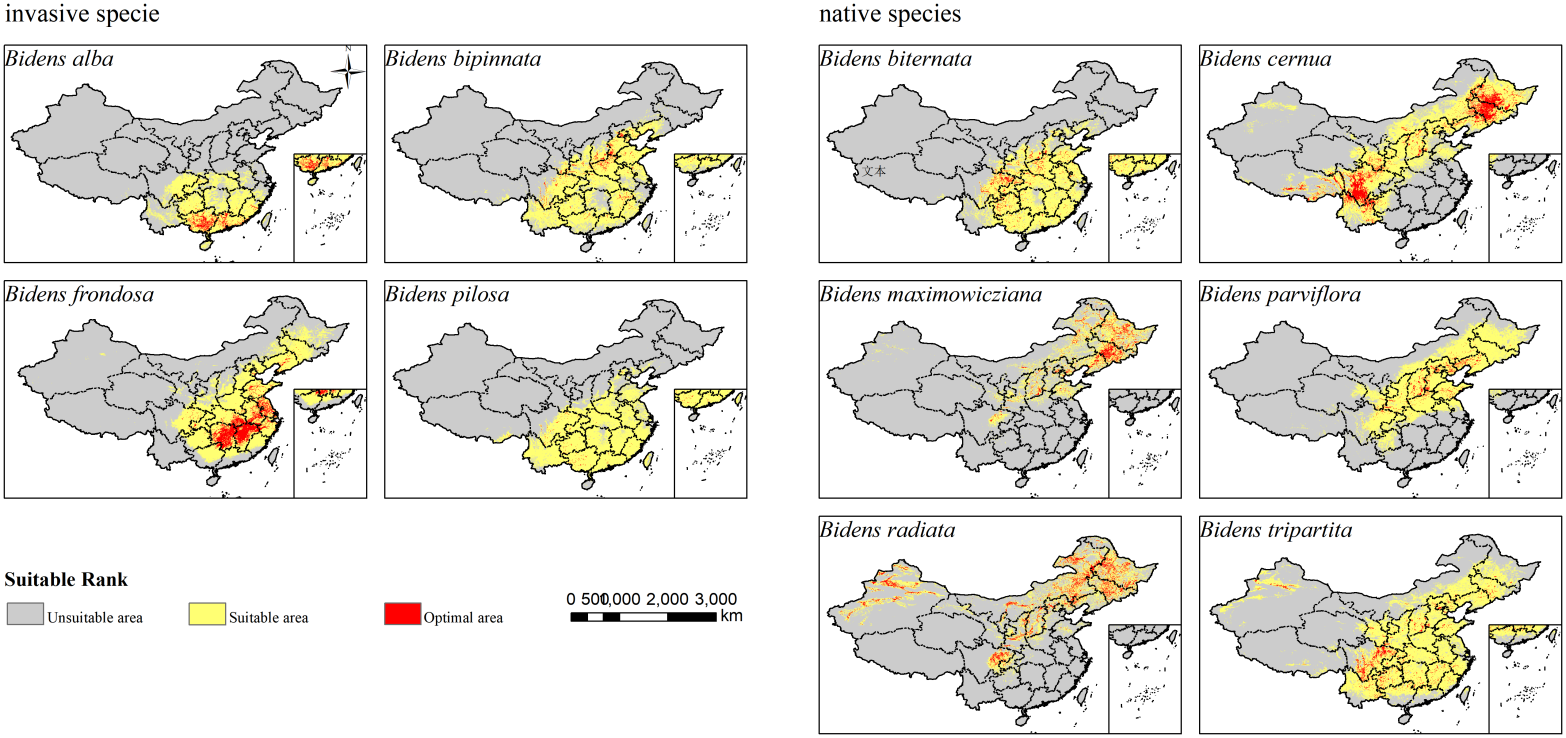
Prediction of potential suitable areas of Bidens genu under current climate model.

Under the current scenario, the potential suitable areas for the four invasive species are mainly in Southern China (**Figure 2**). Among them, *B. frondosa* has the largest potential suitable area of 272.491×10^4^ km^2^, and its highly suitable area is 36.563×10^4^ km^2^, and most species in the highly suitable area of invasive species. The second is *B. bipinnata*, and its potential suitable area is 250.681×10^4^ km^2^, its highly suitable area is 8.362×10^4^ km^2^. The potential suitable area of *B. pilosa* is 242.889×10^4^ km^2^, of which the highly suitable area is 1.876×10^4^ km^2^, which is the least area of highly suitable area among invasive species. The total suitable area of *B. alba* is 145.659×10^4^ km^2^, and the highly suitable area is 9.796×10^4^ km^2^.

Most of the current potential suitable areas for *B. biternata* and *B. tripartite* in native species are concentrated in the Southern region. The potential suitable area of *B. biternata* is 242.321×10^4^ km^2^, of which the highly suitable area is 11.057×10^4^ km^2^. The total area of potential suitable area of *B. tripartite* reach 389.043×10^4^ km^2^, which was the largest area of potential suitable area among the species, and the highly suitable area was 16.501×10^4^ km^2^. The suitable areas of the remaining *B. cernua*, *B. maximowicziana*, *B. parviflora*, *B. radiata* and other species are mostly concentrated in the Northern region, and a small part is in the Northwest and Southwest regions. The potential suitable area of *B. cernua* was 330.793×10^4^ km^2^, of which the highly suitable area accounted for 47.580×10^4^ km^2^. The potential suitable area of *B. maximowicziana* was 143.203×10^4^ km^2^, of which the highly suitable area accounted for 19.012×10^4^ km^2^. The potential suitable area of *B. parviflora* was 260.434×10^4^ km^2^, of which the highly suitable area accounted for 9.456×10^4^ km^2^. The potential suitable area of *B. radiata* is 201.102×10^4^ km^2^, of which the highly suitable area accounts for 37.628×10^4^ km^2^.

### Changes in the spatial distribution range of *Bidens* under different climate models in the future

**Figure 3:**
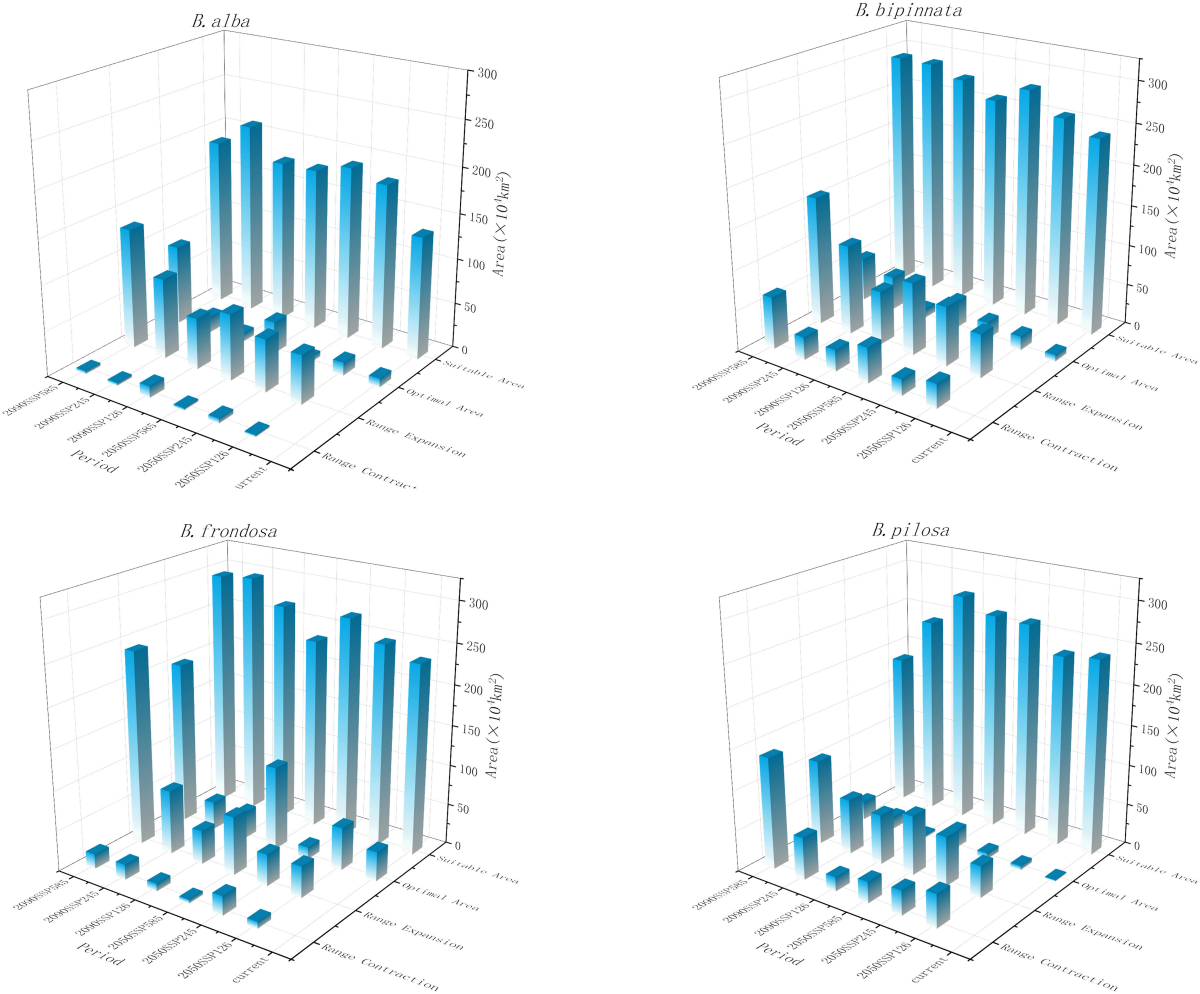
Changes in the spatial distribution range of invasive species of Bidens genu under different climate models in the future.

In the future climate scenario, by the 2090s, the total area of potential suitable areas for the three invasive species of *B. alba*, *B. bipinnata* and *B. frondosa* will show an expanding trend, and the new area will be greater than the lost area (**Figure 3**). Among them, the total area of potential suitable areas for *B. frondosa* increased the most under the 2090 SSP585 scenario, which was 223.252×10^4^ km^2^. The total area of potential suitable area of *B. pilosa* in 2050SSP126 and 2090SSP585 scenarios decreased compared with the current period, losing 1.800×10^4^ km^2^ and 32.672×10^4^ km^2^ respectively, and the total area of potential suitable area increased in the rest periods. The potential suitable areas of the four invasive species mainly expanded to high latitudes, but shrank in low latitudes.

**Figure 4:**
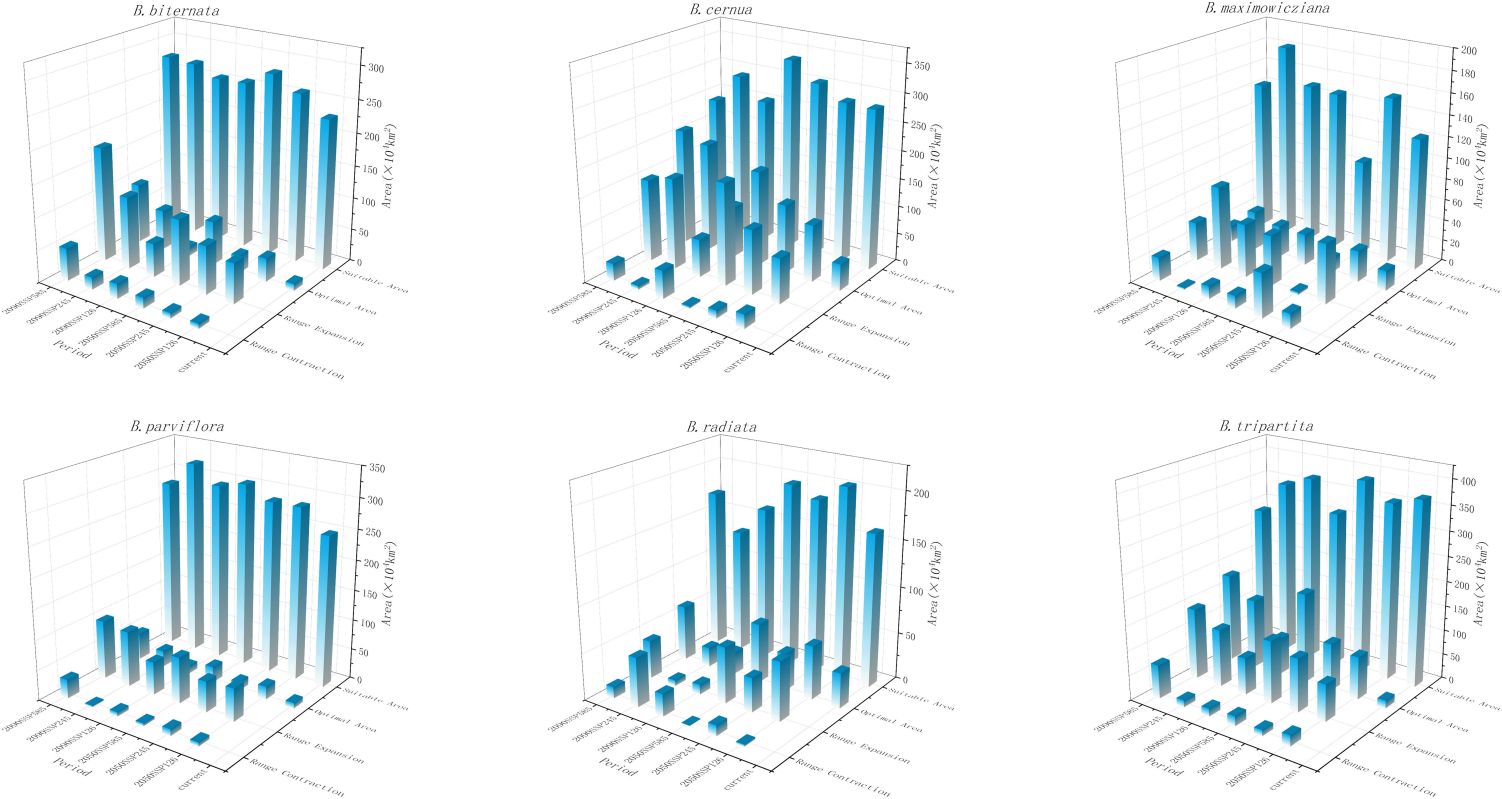
Changes in the spatial distribution range of native species of Bidens genu under different climate models in the future.

Among the six native species, *B. biternata, B. cernua, B. maximowicziana, B. parviflora, B. radiata* and *B. tripartita*. Except that the total potential suitable area of *B. maximowicziana* decreased by 39.264×10^4^ km^2^ under 2050SSP245 scenario and the total potential suitable area of *B. radiata* decreased by 13.201×10^4^ km^2^ and 46.857×10^4^ km^2^ under 2090SSP126 and 2090SSP245 scenarios, the total potential suitable area of other species increased in the future (**Figure 4**). Among them, the new area of *B. cernua* was the largest under 2050SSP585 and 2090SSP245 scenarios. It reached 177.432×10^4^ km^2^ and 155.339×10^4^ km^2^, while the new area in 2090 SSP126 was the least, only 17.463×10^4^ km^2^. The potential suitable area of native species is reflected in the expansion of high latitude area and the contraction of low latitude area, but the suitable area of *B. cernua* and *B. tripartita* in the northwest region is shrinking.

### The change of distribution centroid of potential suitable areas of *Bidens* under future climate change

**Figure 5:**
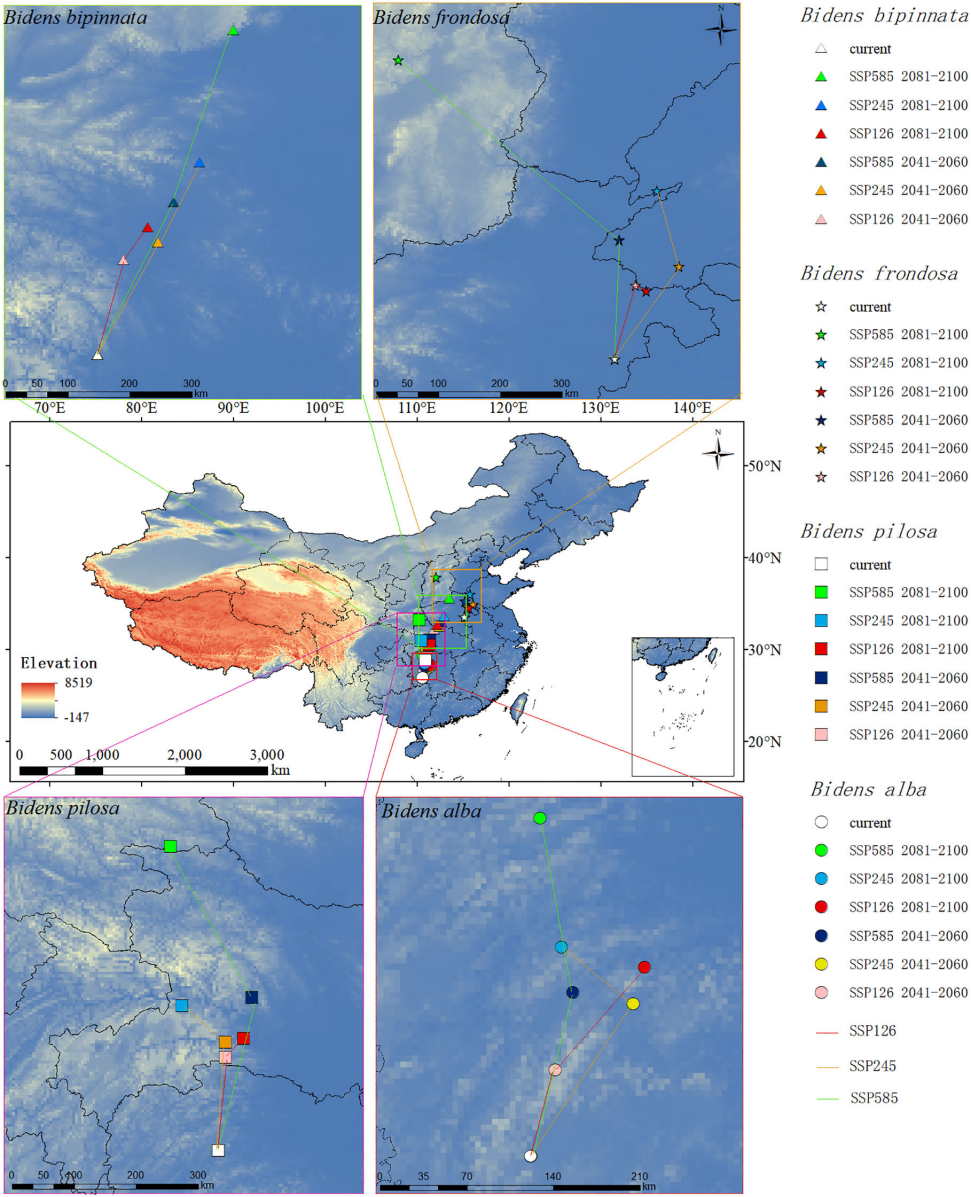
Centroid migration diagram of invasive species distribution.

According to the figure, the migration paths of the potential distribution centroids of the four invasive species of *B. alba, B. bipinnata, B. frondosa*, and *B. pilosa* in the next three scenarios are similar, and they all have a tendency to expand to high latitudes (**Figure 5**). The distribution centroid of *B. alba* migrated was (110°31′43″E, 26°56′22″N). The distribution centroid of *B. bipinnata* migrated was (111°27′31″E, 30°47′23″N. The distribution centroid of *B. frondosa* migrated was (115°07′42″E, 33°32′56″N). The distribution centroid of *B. pilosa* migrated was (110°52′27″E, 28°49′24″N. The dominant environmental factor of these four plants is the precipitation in the warmest quarter, and their migration directions are roughly the same.

**Figure 6:**
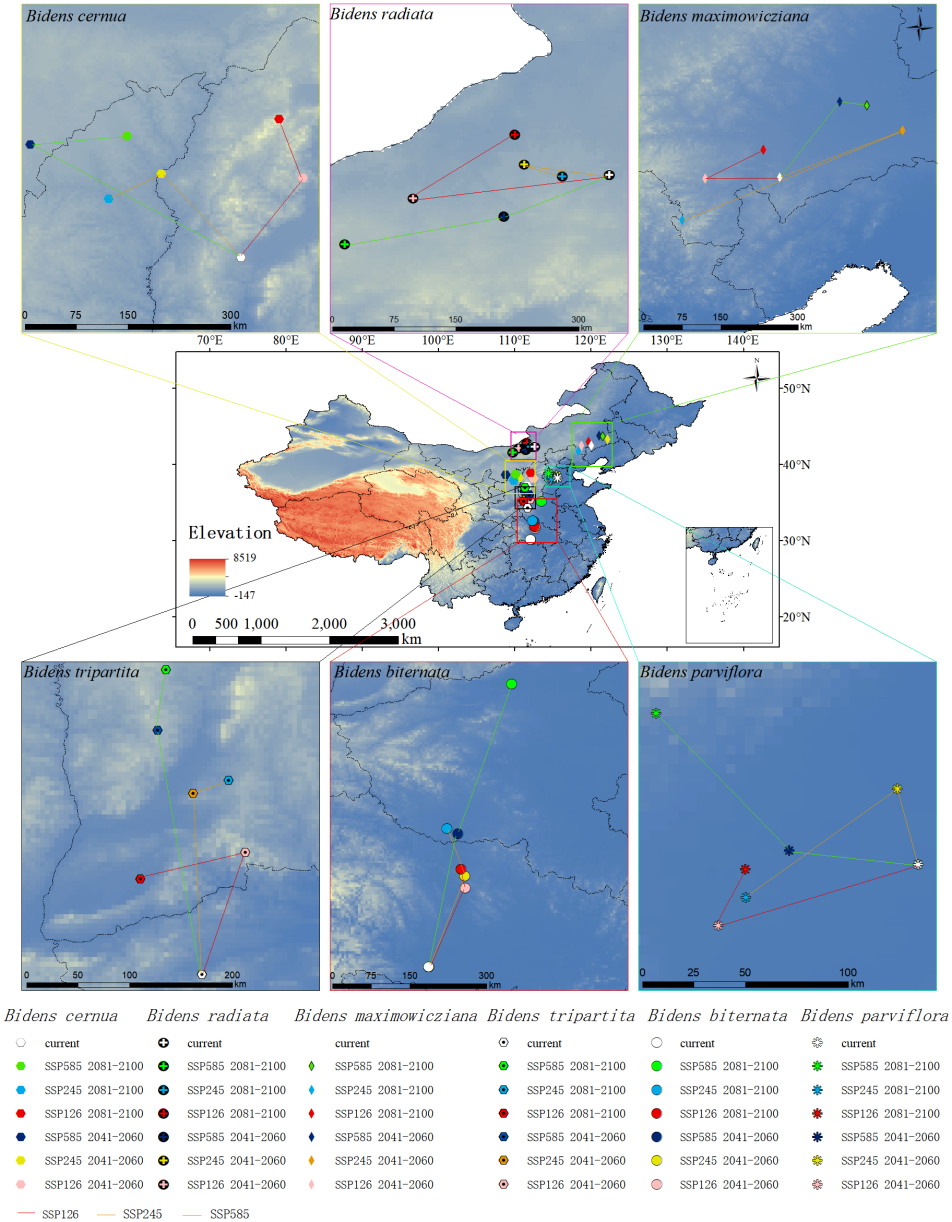
Centroid migration map of native species distribution.

Among the native species, *B. biternata* and *B. tripartita* migrated in the same direction to high latitudes (**Figure 6**). The distribution centroid of *B. biternata* migrated was (112°01′18″E, 30°11′00″N). The distribution centroid of *B. tripartita* migrated was (111°40′06″E, 34°18′10″N). The dominant environmental factor in *B. biternata* is the Precipitation of Warmest Quarter, and its migration direction is mainly related to precipitation, so it migrates northward. The dominant environmental factor of *B. tripartita* is human activity factor, but the Precipitation Seasonality and the contribution rate of Precipitation of Warmest Quarter to *B. tripartita* are also large. Therefore, the trend of *B. tripartita* moving northward is also obvious.

The four species of *B. cernua, B. maximowicziana, B. parviflora* and *B. radata* do not have the same migration direction as the above species. The distribution centroid of *B. cernua* migrated was (111°36′58″E, 37°08′26″N), Under the SSP126 scenario, it moves to the northeast, and under the SSP245 and SSP585 scenarios, it moves to the northwest. The distribution centroid of *B. maximowicziana* migrated was (120°01′44″E, 42°29′39″N), In the SSP126 scenario, it moves northwestward, in the SSP585 scenario, it moves northeastward, and in the SSP245 scenario, it moves northeastward first and then southwestward. The distribution centroid of *B. parviflora* migrated was (115°35′27″E, 38°13′35″N), Under the SSP126 scenario, it migrates to the southwest, under the SSP585 scenario, it migrates to the northwest, and under the SSP245 scenario, it first migrates to the northwest and then to the southwest. The distribution centroid of *B. radiata* migrated was (112°35′37″E, 42°22′20″N), Under the SSP126 scenario, it first migrates to the southwest and then to the northwest. Under the SSP245 scenario, it first migrates to the northwest and then to the southwest. The dominant environmental factors of these four plants are human activity factor, followed by climatic factors. Human activity factor will affect the growth and distribution of species. Therefore, the migration direction of these four plants is not simply moving towards high latitudes.

## Discussion

### Evaluation of predictive ability and contribution rate of maxent model

The MaxEnt model analyzes the distribution of species when the entropy reaches the maximum value under limited conditions. It has many advantages, such as wide application range, high precision, simple operation, low sample quantity requirement, stable operation results, etc (Li et al., 2021). In this study, the MaxEnt model was used for the first time to obtain the potential geographic distribution map of invasive and native species of *Bidens* genu. The model has excellent fitting ability (AUC values are greater than 0.9), indicating that the model can objectively predict the potential suitable growth area of the species. The MaxEnt model was tested by Jacknife and the contribution rate was calculated. The results showed that the Precipitation of Warmest Quarter (bio18) was the most important environmental factor affecting the distribution of invasive species, and human activity factor (HA) was the most important environmental factor affecting the distribution of native species. From the top three environmental factors, it can be found that the environmental factors affecting the survival of *Bidens* genu are mainly precipitation, temperature and human activities, which is consistent with the main ecological factors of most plants (Sun et al., 2017). According to the results of the most suitable threshold value of the dominant factor, the lowest Precipitation of Warmest Quarter suitable for the growth of invasive species is 346.632 mm, and the highest is 1438.008 mm, indicating that invasive species prefer warm and humid climate. The lowest human activity index causing the growth and distribution of native species was 31.416, and the highest was 197.88, indicating that human activities were related to the distribution of native species. Therefore, the environmental change caused by climate change leads to the change of spatial distribution of plants (Zhang et al., 2021a; Tang et al., 2021b; Zhao et al., 2023b). *Bidens* genu are easily adhered to human clothing and livestock fur because of the barbed spines on the top of their fruits. Therefore, human activities can easily affect the distribution of *Bidens* genu and achieve long-distance transmission and diffusion (Ma, 2021).

### Species distribution prediction of *Bidens* under current and future climate scenarios

Adaptation to climate and climate change is essential for plant growth, geographical distribution and biodiversity (Liu et al., 2022a). Precipitation is the main environmental factor affecting the growth and distribution of plants in different habitats (Warren et al., 2021). The MaxEnt model predicts that, the main suitable areas for the four invasive species of *B. alba, B. bipinnata, B. frondosa*, and *B. pilosa* and the native species *B. biternata* in the current climate scenario are East China, Central China, South China, and Southwest China. The climate of these areas humid, and the human disturbance is strong, which is suitable for the colonization and diffusion of invasive plants. Studies have shown that the high suitable areas of *B. alba* are mainly in Guangxi Zhuang and Guangdong Province in China. *B. alba* has a large suitable area in southern China, mainly concentrated in the southern border and coastal provinces, which is consistent with the previous research results of *B. alba* (Yue et al., 2016). The current distribution areas of native species and invasive species are different. The main suitable areas of *B. cernua, B. maximowicziana, B. parviflora, B. radiata* and *B. tripartita* are northeast, north, central and southwest regions. Most of them are temperate monsoon climate, and have high temperature and rainy summer, and cold and dry in winter. Studies have shown that climate change will increase, fluctuate or reduce the distribution range of species (Yuan et al., 2015), The results showed that under the influence of future climate change, the suitable areas of invasive species and native species of *Bidens* genu have increased significantly. However, under the 2050SSP126 and 2090sSSP585 scenarios, the total suitable area of the invasive species *B. pilosa*, the native species *B. maximowicziana* in the 2050SSP245 period, and the *B. radiata* in the 2090SSP126 and 2090SSP245 periods decreased slightly. First, it may be due to changes in temperature and precipitation during this period. The climate is not suitable for the survival of the species. Secondly, the distribution range of *B. maximowicziana* and *B. radiata* is more dispersed, and most of them are scattered in the northern region, indicating they have strict environmental requirements. Under climate change and intense human activities, the distribution area of more dispersed species may be narrowed, while the distribution area of widely distributed species may be expanded (Zhao et al., 2023a; Cao et al., 2021).

### Spatial pattern and centroid distribution changes of *Bidens*

In the future climate, with the exponential growth of global greenhouse gas emissions, the trend of future climate warming in China will be further aggravated (Li et al., 2020b; Guo et al., 2018). Precipitation is an important guarantee to maintain the normal growth and physiological activities of plants (Korell et al., 2021). In this study, the spatial distribution area of all species showed an increase in high latitudes and a decrease in low latitudes. This is because the impact of climate change leads to an increase in environmental pressure in low latitudes, resulting in fewer or extinct species in the region (Parmesan and Hanley, 2015). At the same time, compared with invasive species, the impact of climate change on the distribution pattern of native plants is more complex. The cumulative contribution rate of precipitation factors of the four invasive species is significantly greater than that of other factors, indicating that invasive species have higher requirements for precipitation. In the future, with the global climate warming and the northward movement of precipitation, the suitable area of invasive species in geographical space will expand to the high latitudes of northern China, this, is consistent with the expansion trend of the invasive species of *Bidens* genu, such as *B. frondosa* and *B. Pilosa* (Yue et al., 2016; Du et al., 2021). The suitable area of plants in the high latitudes of native species has increased or decreased. It may be because the dominant factor of native species is human activities, human migration, land use and other activities will lead to plant death or migration (Cao et al., 2022).

At the same time, the distribution centroid of invasive species expands to higher latitudes, which may be due to the gradual increase of rainfall from the current altitude to higher altitudes with greenhouse gas emissions. Therefore, most species will move to higher latitudes and altitudes to adapt to climate change (Jiang et al., 2022). The migration direction of invasive species is consistent with previous research, indicating that climate warming leads to the migration of species to higher latitudes (Liu et al., 2022b; Ye et al., 2020). Compared with invasive species, the migration direction of native species is more complex in the future climate background, because different human activities, methods and intensities will lead to different types of land use (cultivated land, forest land, shrub land and residential land), which directly affects the spatial distribution and diversity of plants (Zhao et al., 2023a), Therefore, local species use human activity factor as predictors, which can more accurately show the future potential distribution range and spatial pattern of local species. The migration path of native species with human activity factor as the dominant factor is more complex than that of invasive species. The distribution centroids of *B. maximowicziana* in 2090 SSP245 period, *B. radiata* in 2050 and 90 SSP585 periods, and *B. parviflora* in 2050 and 90 SSP126 periods all migrated to lower latitudes than at present. This is due to the dual effects of climate and human activities. Even in areas with suitable climate, the reduction of natural habitats caused by human is overuse of land which hinder the expansion of species suitable areas (Liu et al., 2021). According to the prediction results, the dominant environmental factor of the five plants in the local species is human activity, Further, the closer distribution center of mass is to the northwest region, the smaller the migration range and the more complex the direction. Because the population in the northern region is scarce, the spread of plant seeds depends on human activities. While human activities promote the spread of plant seeds, the scope, mode and intensity of human activities will also hinder the expansion of species (Zhang et al., 2022b).

### Summary and recommendations

In this study, the current distribution data of species were used for model analysis, and the prediction model was used to predict the future distribution area of species, The model predicted the invasion trend of invasive species in advance and establish a prevention mechanism, and establish a local species protection scheme. However, the model prediction was based on the current species distribution data and the future prediction of climate change. Therefore, the specific distribution of future species may be different from the prediction. According to the change of the distribution range of species, the establishment of invasive species prevention and local species protection mechanism, put forward the following suggestions: Firstly, establish an invasive species protection zone in the future suitable areas predicted by the invasive species *B. alba*, *B. bipinnata*, *B. frondosa*, and *B. pilosa* to prevent the continued spread of invasive species. Secondly, establish local species reserves in areas where the predicted future suitable areas for local species are reduced to protect local species plant resources. Thirdly, strengthen the public awareness of invasive species prevention and guide residents to strengthen the protection of native species.

## Conclusion

This study explored the effects of climate change on the distribution of invasive and native species of *Bidens*. The results showed that the suitable areas for invasive species were mainly located in southern China, while the suitable areas for native species were mainly located in northern China. In the future climate scenarios, the potential suitable areas of all species will be transferred to high latitudes. Precipitation in the warmest quarter and human activities were the main environmental factors affecting the potential distribution of invasive species and native species, respectively. In summary, this study provides strong evidence for the potential suitable area distribution of invasive and native species of *Bidens* under the background of climate change. This will become a useful reference for preventing malignant invasion of invasive species and establishing protection mechanisms for native species. It also fills the gap in the future distribution pattern change of *Bidens* in China.

## Data Availability

The raw data supporting the conclusions of this article will be made available by the authors, without undue reservation.

## References

[B1] Barbosa F. G. Schneck F. (2015). Characteristics of the top-cited papers in species distribution predictive models. Ecol. Model. 313, 77–83. doi: 10.1016/j.ecolmodel.2015.06.014

[B2] Brandão M. Krettli A. Soares L. Nery C. Marinuzzi H.. (1997). Antimalarial activity of extracts and fractions from Bidens pilosa and other *Bidens* species (Asteraceae) correlated with the presence of acetylene and flavonoid compounds. J. ethnopharmacol 57, 131–138. doi: 10.1016/S0378-8741(97)00060-3 9254115

[B3] Brändel M. (2004). “Dormancy and germination of heteromorphic achenes of *Bidens frondosa* ,” in Flora-Morphology, Distribution, Functional Ecology of Plants, vol. 199. , 228–233. doi: 10.1078/0367-2530-00150

[B4] Byeon D. H. Jung S. Lee W. H. (2018). Review of CLIMEX and MaxEnt for studying species distribution in South Korea. J. Asia Pac Biodivers 11, 325–333. doi: 10.1016/j.japb.2018.06.002

[B5] Cao X. Y. Tian F. Herzschuh U. Ni J. Xu Q. H. Li W. J.. (2022). Human activities have reduced plant diversity in eastern China over the last two millennia. Global Change Biol. 28, 4962–4976. doi: 10.1111/gcb.16274 35596650

[B6] Cao W. Wu D. Huang L. Pan M. Huhe T. (2021). Determinizing the contributions of human activities and climate change on greening in the Beijing–Tianjin–Hebei Region, China. Sci. Rep. 11, 21201. doi: 10.1038/s41598-021-00788-4 34707210 PMC8551181

[B7] Cao Y. S. Xiao Y. A. Zhang S. S. Hu W. H. (2018). Simulated warming enhances biological invasion of *Solidago canadensis* and *Bidens frondosa* by increasing reproductive investment and altering flowering phenology pattern. Sci. Rep. 8, 16073. doi: 10.1038/s41598-018-34218-9 30375415 PMC6207732

[B8] Chiang Y. M. Chuang D. Y. Wang S. Y. Kuo Y. H. Tsai P. W. Shyur L. F. (2004). Metabolite profiling and chemopreventive bioactivity of plant extracts from *Bidens pilosa* . J. ethnopharmacol 95, 409–419. doi: 10.1016/j.jep.2004.08.010 15507368

[B9] Cook J. Nuccitelli D. Green S. A. Richardson M. Winkler B. Painting R.. (2013). Quantifying the consensus on anthropogenic global warming in the scientific literature. Environ. Res. Lett. 8, 24024. doi: 10.1088/1748-9326/8/2/024024

[B10] Deng Q. Deng Q. X. Wang Y. Li L. Long X. Y. Ren S.. (2019). Effects of intercropping with *Bidens* species plants on the growth and cadmium accumulation of *Ziziphus acidojujuba* seedlings. Environ. Monit Assess. 191, 1–8. doi: 10.1007/s10661-019-7375-6 31053931

[B11] Dong R. Hua L. M. Hua R. Ye G. H. Bao D. H. Cai X. C.. (2023). Prediction of the potentially suitable areas of *Ligularia virgaurea* and *Ligularia sagitta* on the Qinghai–Tibet Plateau based on future climate change using the MaxEnt model. Front. Plant Sci. 14. doi: 10.3389/fpls.2023.1193690 PMC10400714 37546265

[B12] Du Z. X. Su Q. T. Zhou B. Yan X. H. Li X. H. Xiao Y. A. (2021). Potential distribution of invasive species *Bidens frondosa* under different climate change scenarios in China. Chin. J. Ecol. 40, 2575–2582. doi: 10.13292/j.1000-4890.202109.023

[B13] Fick S. E. Hijmans R. J. (2017). WorldClim 2: New 1-km spatial resolution climate surfaces for global land areas. Int. J. Climatology. 37, 4302–4315. doi: 10.1002/joc.5086

[B14] Fotso A. F. Longo F. Djomeni D. D. Kouam S. F. Spiteller M. Dongmo A. B.. (2014). Analgesic and antiinflammatory activities of the ethyl acetate fraction of *Bidens pilosa* (Asteraceae). Inflammopharmacology 22, 105–114. doi: 10.1007/s10787-013-0196-2 24242914

[B15] Guo F. Lenoir J. Bonebrake T. C. (2018). Land-use change interacts with climate to determine elevational species redistribution. Nat. Commun. 9, 1315. doi: 10.1038/s41467-018-03786-9 29615626 PMC5883048

[B16] Hayat U. Shi J. Wu Z. J. Rizwan M. Haider M. S. (2024). Which SDM Model, CLIMEX vs. MaxEnt, Best Forecasts Aeolesthes sarta Distribution at a Global Scale under Climate Change Scenarios? Insects 15, 324. doi: 10.3390/insects15050324 38786880 PMC11121915

[B17] Hirzel A. H. Hausser J. Chessel D. Perrin N. (2002). Ecological-niche factor analysis: how to compute habitat-suitability maps without absence data? Ecology 83, 2027–2036. doi: 10.1890/0012-9658(2002)083

[B18] Hu H. W. Wei Y. Q. Wang W. Y. Suonan J. Wang S. X. Chen Z.. (2022). Richness and distribution of endangered orchid species under different climate scenarios on the Qinghai-Tibetan Plateau. Front. Plant Sci. 13. doi: 10.3389/fpls.2022.948189 PMC9490128 36160966

[B19] Jiang R. P. Zou M. Qin Y. Tan G. D. Huang S. P. Quan H. G.. (2022). Modeling of the potential geographical distribution of three *Fritillaria* species under climate change. Front. Plant Sci. 12. doi: 10.3389/fpls.2021.749838 PMC8784777 35082804

[B20] Korell L. Auge H. Chase J. M. Harpole W. S. Knight T. M. (2021). Responses of plant diversity to precipitation change are strongest at local spatial scales and in drylands. Nat. Commun. 12, 2489. doi: 10.1038/s41467-021-22766-0 33941779 PMC8093425

[B21] Li J. J. Fan G. He Y. (2020a). Predicting the current and future distribution of three Coptis herbs in China under climate change conditions, using the MaxEnt model and chemical analysis. Sci. Total Environ. 698, 134141. doi: 10.1016/j.scitotenv.2019.134141 31505366

[B22] Li Y. C. Li M. Y. Li C. Liu Z. Z. (2020b). Optimized maxent model predictions of climate change impacts on the suitable distribution of *cunninghamia lanceolata* in China. Forests 11, 302. doi: 10.3390/f11030302

[B23] Li Y. H. Zhang L. J. Zhu W. B. Zhang J. J. Xu S. B. Zhu L. Q. (2021). Changes of *Taxus chinensis* var. *mairei* habitat distribution under global climate change. J. Natural Reso 36, 783–792. doi: 10.31497/zrzyxb.20210318

[B24] Liu Y. An D. S. Xu D. D. Zhu J. Q. (2022b). Review on the vegetation response to climate change in vertical zone spectrum. Ecol. Sci. 41, 245–251. doi: 10.14108/j.cnki.1008-8873.2022.03.029

[B25] Liu C. Huo H. L. Tian L. M. Dongm X. G. Xu J. Y. Qi D.. (2020). Prediction of potential geographical distribution patterns of *Pyrus xerophila* under different climate scenarios. Chin. J. Appl. Ecol. 31, 4073–4079. doi: 10.13287/j.1001-9332.202012.012 33393244

[B26] Liu H. R. Khan G. Gao Q. B. Zhang F. Q. Liu W. H. Wang Y. F.. (2022a). Dispersal into the Qinghai–Tibet plateau: evidence from the genetic structure and demography of the alpine plant Triosteum pinnatifidum. PeerJ 10, e12754. doi: 10.7717/peerj.12754 35178292 PMC8815373

[B27] Liu L. Zhang Y. Y. Huang Y. Zhang J. D. Mou Q. Y. Qiu J. Y.. (2021). Simulation of potential suitable distribution of original species of Fritillariae Cirrhosae Bulbus in China under climate change scenarios. Environm Sci. pollut. R 29, 22237–22250. doi: 10.1007/s11356-021-17338-0 34780014

[B28] Ma B. (2021). A study of Bidens L. (Asteraceae) in China based on morphology and molecular phylogeny. Zhengzhou University. doi: 10.27466/d.cnki.gzzdu.2021.002218

[B29] Meng Y. Ma J. M. Wang Y. Q. Mo Y. H. (2020). Prediction of distribution area of *Loropetalum chinense* based on Maxent model. Acta Ecologica Sin. 40, 8287–8296. doi: 10.5846/stxb201911252549

[B30] Merow C. Smith M. J. Silander J. A. (2013). A practical guide to MaxEnt for modeling species’ distributions: what it does, and why inputs and settings matter. Ecography 36, 1058–1069. doi: 10.1111/j.1600-0587.2013.07872.x

[B31] Neftalí S. Salvador A. C. Urtzi E. U. Cândida G. V. Diana S. G. Fernando M. F.. (2021). Want to model a species niche? A step-by-step guideline on correlative ecological niche modelling. Ecol. Model. 456, 109671. doi: 10.1016/j.ecolmodel.2021.109671

[B32] Nelder J. A. Wedderburn R. W. (1972). Generalized linear models. J. R Stat. Soc. A 135, 370–384. doi: 10.2307/2344614

[B33] Nikendel C. Bugaj T. J. Nikendel F. Kühl S. J. Kühl M. (2020). Climate change: Causes, consequences, solutions and public health care implications. Z. Evid. Fortbild. Qual. Gesundh. wesen (ZEFQ) 156, 59–67. doi: 10.1016/j.zefq.2020.07.008 32859556

[B34] Ouyang X. H. Bai S. H. Strachan G. B. Chen A. L. (2022). Simulation of the potential distribution of rare and endangered *Satyrium* species in China under climate change. Ecol. Evol. 12, e9054. doi: 10.1002/ece3.9054 35845387 PMC9273742

[B35] Parmesan C. Hanley M. E. (2015). Plants and climate change: complexities and surprises. Ann. bot-london 116, 849–864. doi: 10.1093/aob/mcv169 PMC4640131 26555281

[B36] Phillips S. J. Anderson R. P. Schapire R. E. (2006). Maximum entropy modeling of species geographic distributions. Ecol. Model. 190, 231–259. doi: 10.1016/j.ecolmodel.2005.03.026

[B37] Qiang S. Zhang H. (2022). Invasion and management of alien plants in agroecosystems in China. J. Nanjing Agric. Univ. 45, 957–980. doi: 10.7685/jnau.202206010

[B38] Qiu L. Jacquemyn H. Burgess K. S. Zhang L. G. Zhou Y. D. Yang B. Y.. (2023). Contrasting range changes of terrestrial orchids under future climate change in China. SciTotal Environ. 895, 165128. doi: 10.1016/j.scitotenv.2023.165128 37364836

[B39] Rew L. J. Brummer T. J. Pollnac F. W. Larson C. D. Taylor K. T. Taper M. L.. (2018). Hitching a ride: Seed accrual rates on different types of vehicles. J. Environ. manage 206, 547–555. doi: 10.1016/j.jenvman.2017.10.060 29127927

[B40] Salgado V. G. Viera Barreto J. N. Rodríguez-Cravero J. F. Grossi M. A. Gutiérrez D. G. (2024). Factors influencing the global invasion of the South American weedy species *Praxelis clematidea* (*Asteraceae)*: a niche shift and modelling-based approach. Bot. J. Lin Soc. boae079. doi: 10.1093/botlinnean/boae079

[B41] Shi Y. H. Ren Z. G. Wang W. J. Xu X. Liu J. Zhao Y. H.. (2021). Predicting the spatial distribution of three Astragalusspecies and their pollinating bumblebees in the Sino-Himalayas. Biodivers Sci. 29, 759. doi: 10.17520/biods.2020268

[B42] Stockwell D. R. Noble I. R. (1992). Induction of sets of rules from animal distribution data: a robust and informative method of data analysis. Math Comput. simulat 33, 385–390. doi: 10.1016/0378-4754(92)90126-2

[B43] Su Q. T. Du Z. X. Luo Y. Zhou B. Xiao Y. A. Zou Z. R. (2024b). MaxEnt Modeling for Predicting the Potential Geographical Distribution of *Hydrocera triflora* since the Last Interglacial and under Future Climate Scenarios. Biology 13, 745. doi: 10.3390/biology13090745 39336172 PMC11428515

[B44] Su Q. T. Du Z. X. Xue Y. X. Li H. (2024a). Habitat suitability modeling of endemic genus chimonanthus in China under climate change. Forests 15, 1625. doi: 10.3390/f15091625

[B45] Sun H. Wang X. P. Fan Y. W. Liu C. Wu P. Li Q. Y.. (2017). Effects of biophysical constraints, climate and phylogeny on forest shrub allometries along an altitudinal gradient in Northeast China. Sci. Rep. 7, 43769. doi: 10.1038/srep43769 28266604 PMC5339776

[B46] Tang X. G. Yuan Y. D. LI X. M. Zhang J. C. (2021a). Maximum entropy modeling to predict the impact of climate change on pine wilt disease in China. Front. Plant Sci. 12. doi: 10.3389/fpls.2021.652500 PMC8102737 33968109

[B47] Tang X. G. Yuan Y. D. Wang L. J. Chen S. R. Liu X. Zhang J. C. (2021b). Identifying prioritized planting areas for medicinal plant Thesium chinense Turcz. under climate change in China. Ecol. Inform 66, 101459. doi: 10.1016/j.ecoinf.2021.101459

[B48] Wang Y. Lian J. H. Shen H. Zhang R. Y. Guo Y. Ye W. H. (2020). The effects of *Bidens alba* invasion on soil bacterial communities across different coastal ecosystem land-use types in southern China. PloS One 15, e0238478. doi: 10.1371/journal.pone.0238478 33112879 PMC7592744

[B49] Wang S. Y. Lu Y. Y. Han M. Y. Li L. L. He P. Shi A. M.. (2023). Using MaxEnt model to predict the potential distribution of three potentially invasive scarab beetles in China. Insects 14, 239. doi: 10.3390/insects14030239 36975924 PMC10054099

[B50] Wang R. Tong L. Liu C. Y. Shi Y. P. (2018). Research progress of polyacetylenes from *Bidens* genus plants and their biological activity. Chin. Tradit Herbal Drugs 49, 4189–4196. doi: 10.7501/j.issn.0253-2670.2018.17.034

[B51] Warren D. L. Dornburg A. Zapfe K. Iglesias T. L. (2021). The effects of climate change on Australia’s only endemic Pokémon: Measuring bias in species distribution models. Methods Ecol. Evol. 12, 985–995. doi: 10.1111/2041-210X.13591

[B52] Warren D. L. Glor R. E. Turelli M. (2010). ENMTools: a toolbox for comparative studies of environmental niche models. Ecography 33, 607–611. doi: 10.1111/j.1600-0587.2009.06142.x

[B53] Wilson R. J. Gutierrez D. Gutierrez J. Monserrat V. J. (2007). An elevational shift in butterfly species richness and composition accompanying recent climate change. Global Change Biol. 13, 1873–1887. doi: 10.1111/j.1365-2486.2007.01418.x

[B54] Xu L. Fan Y. Zheng J. H. Guan J. Y. Lin J. Wu J. G.. (2024). Impacts of climate change and human activity on the potential distribution of *Aconitum leucostomum* in China. Sci. Total Environ. 912, 168829. doi: 10.1016/j.scitotenv.2023.168829 38030008

[B55] Xu Y. Zhu R. Gao L. Huang D. Fan Y. Liu C.. (2023). Predicting the current and future distributions of *Pennisetum alopecuroides* (L.) in China under climate change based on the MaxEnt model. PloS One 18, e0281254. doi: 10.1371/journal.pone.0281254 37014870 PMC10072476

[B56] Yang W. Y. Sun S. X. Wang N. X. Fan P. X. Chao Y C. Wang R. Q.. (2023). Dynamics of the distribution of invasive alien plants (Asteraceae) in China under climate change. Sci. Total Environ. 903, 166260. doi: 10.1016/j.scitotenv.2023.166260 37579809

[B57] Ye X. Z. Zhao G. H. Zhang M. Z. Cui X. Y. Fan H. H. Liu B. (2020). Distribution pattern of endangered plant *Semiliquidambar cathayensis* (Hamamelidaceae) in response to climate change after the last interglacial period. Forests 11, 434. doi: 10.3390/f11040434

[B58] Yuan H. S. Wei Y. L. Wang X. G. (2015). Maxent modeling for predicting the potential distribution of Sanghuang, an important group of medicinal fungi in China. Fungal Ecol. 17, 140–145. doi: 10.1016/j.funeco.2015.06.001

[B59] Yue M. F. Feng L. Cui Y. Zhang C. Tian X. S. (2016). Prediction of the potential distribution and suitability analysis of the invasive weed,*Bidens alba* (L.) DC. J. biosafety 25, 222–228. doi: 103969/j.issn.2095-1787.2016.03.013

[B60] Zhang W. Chen X. Liu R. Song X. Liu G. Zou J.. (2022a). Realized niche shift associated with Galinsoga quadriradiata (Asteraceae) invasion in China. J. Plant Ecol. 15, 538–548. doi: 10.1093/jpe/rtab086

[B61] Zhang Y. C. Jiang X. H. Lei Y. X. Wu Q. L. Liu Y. H. Shi X. W. (2023). Potentially suitable distribution areas of *Populus euphratica* and *Tamarix chinensis* by MaxEnt and random forest model in the lower reaches of the Heihe River, China. Environ. Monit Assess. 195, 1519. doi: 10.1007/s10661-023-12122-8 37993760

[B62] Zhang S. Liu X. G. Li R. M. Wang X. L. Cheng J. H. Yang Q. L.. (2021a). AHP-GIS and MaxEnt for delineation of potential distribution of Arabica coffee plantation under future climate in Yunnan, China. Ecol. indic 132, 108339. doi: 10.1016/j.ecolind.2021.108339

[B63] Zhang Y. B. Meng Q. X. Wang Y. Z. Zhang X. L. Wang W. (2020). Climate change-induced migration patterns and extinction risks of Theaceae species in China. Ecol. Evol. 10, 4352–4361. doi: 10.1002/ece3.6202 32489602 PMC7246209

[B64] Zhang L. X. Sun Z. Y. An B. Zhang D. X. Chen L. Y. (2022b). Alpine musk deer (*Moschus chrysogaster*) adjusts to a human-dominated semi-arid mountain ecosystem. Animals-basel 12, 3061. doi: 10.3390/ani12213061 36359183 PMC9658949

[B65] Zhang Y. Tang J. S. Ren G. Zhao K. X. Wang X. F. (2021b). Global potential distribution prediction of *Xanthium italicum* based on Maxent model. Sci. Rep. 11, 16545. doi: 10.1038/s41598-021-96041-z 34400696 PMC8368065

[B66] Zhang Y. Wan Y. Wang C. Chen J. Si Q. Ma F. (2024). Potential distribution of three invasive agricultural pests in China under climate change. Sci. Rep. 14, 13672. doi: 10.1038/s41598-024-63553-3 38871779 PMC11176333

[B67] Zhao D. Wang J. Dai W. Ye K. H. Chen J. Lai Q. L.. (2023a). Effects of climate warming and human activities on the distribution patterns of *Fritillaria unibracteata* in eastern Qinghai-Tibetan Plateau. Sci. Rep. 13, 15770. doi: 10.1038/s41598-023-42988-0 37737302 PMC10516939

[B68] Zhao Y. H. Wen Y. F. Zhang W. Q. Wang C. C. Yan Y. D. Hao S. W.. (2023b). Distribution pattern and change prediction of *Phellodendron* habitat in China under climate change. Ecol. Evol. 13, e10374. doi: 10.1002/ece3.10374 37636866 PMC10450841

