# Supplementary material for: Differences in responses of invasive and native plants to climate change: a case study of Bidens (Asteracea) from China
Source: Front Plant Sci. 2025 Jun 30;16:1583552. doi: 10.3389/fpls.2025.1583552 (PMC12256235; doi:10.3389/fpls.2025.1583552)
Supplement: Supplementary file 1 [file Table1.doc]

**Supplementary Data for**

**Differences in responses of invasive and native plants to climate change: A case study of *Bidens***

Hai-yan Xiao^1^, Da Liao^1^, Shu-jian Zhang^1^, Yu-xin Zhang^1^, Rehab Omer Elnour^2^, Jian-jun Zeng^1^, Xiao-hong Yan^1, *^, Qi-tao Su^1, *^, Bing Zhou^1, *^

^1^School of Life Sciences, Key Laboratory of Jiangxi Province for Biological Invasion and Biosecurity, Jinggangshan University, Ji’an, 343009, China

^2^King Khalid University, Applied College, Dhahran Aljanob, 64261, Saudi Arabia

^*^Corresponding author: Xiao-Hong Yan, yanxiaohong@jgsu.edu.cn; Qi-Tao Su, suqitao@jgsu.edu.cn; Bing Zhou, zhoubing@jgsu.edu.cn

**Table S1.** Potential distribution area and range changes (10^4^ km^2^) of *Bidens* species from the current period to future period (2050s,2090s) under three climate

| classification | species | Period | Range Contraction | Range Expansion | Optimal Area | Suitable Area |
| --- | --- | --- | --- | --- | --- | --- |
| invasive specie | *B.alba* | current | - | - | 9.796 | 135.863 |
|  |  | 2050SSP126 | 2.237 | 54.025 | 14.729 | 182.718 |
|  |  | 2050SSP245 | 5.474 | 58.316 | 6.342 | 192.159 |
|  |  | 2050SSP585 | 2.798 | 73.124 | 35.551 | 180.435 |
|  |  | 2090SSP126 | 12.785 | 56.703 | 9.479 | 180.099 |
|  |  | 2090SSP245 | 3.322 | 88.680 | 18.025 | 212.997 |
|  |  | 2090SSP585 | 3.445 | 133.060 | 88.700 | 186.575 |
|  | *B.bipinnata* | current | - | - | 8.362 | 242.319 |
|  |  | 2050SSP126 | 29.874 | 53.888 | 17.507 | 257.188 |
|  |  | 2050SSP245 | 20.406 | 73.717 | 21.489 | 282.504 |
|  |  | 2050SSP585 | 43.876 | 89.693 | 34.453 | 262.045 |
|  |  | 2090SSP126 | 26.739 | 66.453 | 11.176 | 279.219 |
|  |  | 2090SSP245 | 28.426 | 111.226 | 42.394 | 291.087 |
|  |  | 2090SSP585 | 65.066 | 160.274 | 54.101 | 291.789 |
|  | *B.frondosa* | current | - | - | 36.563 | 235.928 |
|  |  | 2050SSP126 | 8.560 | 39.037 | 53.128 | 249.840 |
|  |  | 2050SSP245 | 23.981 | 39.670 | 15.504 | 272.677 |
|  |  | 2050SSP585 | 4.714 | 72.552 | 103.914 | 236.415 |
|  |  | 2090SSP126 | 9.725 | 42.118 | 34.106 | 270.778 |
|  |  | 2090SSP245 | 18.998 | 79.701 | 35.185 | 298.010 |
|  |  | 2090SSP585 | 18.264 | 241.516 | 202.025 | 293.719 |
|  | *B.pilosa* | current | - | - | 1.876 | 241.013 |
|  |  | 2050SSP126 | 41.387 | 39.587 | 5.510 | 235.580 |
|  |  | 2050SSP245 | 30.682 | 61.071 | 8.045 | 265.233 |
|  |  | 2050SSP585 | 26.516 | 73.754 | 22.803 | 267.324 |
|  |  | 2090SSP126 | 17.872 | 61.756 | 3.565 | 283.208 |
|  |  | 2090SSP245 | 52.535 | 68.148 | 15.655 | 242.848 |
|  |  | 2090SSP585 | 138.107 | 105.435 | 23.750 | 186.467 |
| native species | *B.biternata* | current | - | - | 11.057 | 231.264 |
|  |  | 2050SSP126 | 8.154 | 62.834 | 36.830 | 260.171 |
|  |  | 2050SSP245 | 8.716 | 75.002 | 27.512 | 281.095 |
|  |  | 2050SSP585 | 16.706 | 102.513 | 68.890 | 259.238 |
|  |  | 2090SSP126 | 23.281 | 52.499 | 14.308 | 257.759 |
|  |  | 2090SSP245 | 19.329 | 112.597 | 62.590 | 272.999 |
|  |  | 2090SSP585 | 50.661 | 178.041 | 93.282 | 276.418 |
|  | *B.cernua* | current | - | - | 47.580 | 283.213 |
|  |  | 2050SSP126 | 25.151 | 80.481 | 101.641 | 284.482 |
|  |  | 2050SSP245 | 14.431 | 114.679 | 123.427 | 307.615 |
|  |  | 2050SSP585 | 4.024 | 181.456 | 169.257 | 338.968 |
|  |  | 2090SSP126 | 50.331 | 67.795 | 91.989 | 256.268 |
|  |  | 2090SSP245 | 7.268 | 162.608 | 193.846 | 292.286 |
|  |  | 2090SSP585 | 30.918 | 147.341 | 208.227 | 238.990 |
|  | *B.maximowicziana* | current | - | - | 19.012 | 124.191 |
|  |  | 2050SSP126 | 14.080 | 56.329 | 29.709 | 155.742 |
|  |  | 2050SSP245 | 42.011 | 2.746 | 14.086 | 89.853 |
|  |  | 2050SSP585 | 12.088 | 47.786 | 29.923 | 148.978 |
|  |  | 2090SSP126 | 12.071 | 50.463 | 30.385 | 151.209 |
|  |  | 2090SSP245 | 1.741 | 78.822 | 36.981 | 183.303 |
|  |  | 2090SSP585 | 22.415 | 37.149 | 15.454 | 142.483 |
|  | *B.parviflora* | current | - | - | 9.456 | 250.978 |
|  |  | 2050SSP126 | 6.941 | 55.034 | 21.938 | 286.589 |
|  |  | 2050SSP245 | 11.579 | 51.589 | 14.621 | 285.823 |
|  |  | 2050SSP585 | 4.825 | 76.755 | 26.783 | 305.581 |
|  |  | 2090SSP126 | 7.461 | 54.890 | 13.040 | 294.823 |
|  |  | 2090SSP245 | 2.724 | 92.519 | 27.266 | 322.963 |
|  |  | 2090SSP585 | 31.564 | 97.901 | 44.934 | 281.837 |
|  | *B.radiata* | current | - | - | 37.628 | 163.475 |
|  |  | 2050SSP126 | 2.251 | 63.195 | 57.523 | 204.523 |
|  |  | 2050SSP245 | 11.447 | 36.929 | 40.865 | 185.720 |
|  |  | 2050SSP585 | 0.905 | 60.261 | 64.244 | 196.215 |
|  |  | 2090SSP126 | 24.085 | 10.883 | 24.064 | 163.837 |
|  |  | 2090SSP245 | 53.170 | 6.313 | 21.108 | 133.138 |
|  |  | 2090SSP585 | 12.253 | 41.106 | 59.770 | 170.185 |
|  | *B.tripartita* | current | - | - | 16.501 | 372.542 |
|  |  | 2050SSP126 | 21.888 | 74.798 | 86.617 | 355.336 |
|  |  | 2050SSP245 | 12.418 | 108.262 | 96.809 | 388.079 |
|  |  | 2050SSP585 | 19.963 | 126.109 | 182.514 | 312.675 |
|  |  | 2090SSP126 | 16.173 | 74.695 | 75.708 | 371.857 |
|  |  | 2090SSP245 | 14.358 | 116.356 | 140.444 | 350.597 |
|  |  | 2090SSP585 | 67.724 | 143.464 | 178.611 | 286.173 |
